# Impact of different unconditional monetary incentives on survey response rates in men with prostate cancer: a 2-arm randomised trial

**DOI:** 10.1186/s12874-022-01729-z

**Published:** 2022-09-29

**Authors:** Megan McIntosh, Melissa J. Opozda, Michael O’Callaghan, Andrew D. Vincent, Daniel A. Galvão, Camille E. Short

**Affiliations:** 1 University of Adelaide, Adelaide Medical School, Adelaide, South Australia Australia; 2 Freemasons Centre for Male Health and Wellbeing, South Australian Health and Medical Research Institute and The University of Adelaide, Adelaide, South Australia Australia; 3 Flinders Medical Centre, South Australian Prostate Cancer Clinical Outcomes Collaborative, Adelaide, South Australia Australia; 4 Edith Cowan University, Exercise Medicine Research Institute, Perth, WA Australia; 5 University of Melbourne, Melbourne School of Psychological Sciences and Melbourne School of Health Sciences, Parkville, VIC Australia

**Keywords:** Prostate cancer, Study within a trial, Incentives, Engagement, Survey

## Abstract

**Background:**

Men are often viewed as a difficult group to recruit for psychological research, including in psycho-oncology. Whilst research has demonstrated the effectiveness of small monetary incentives for encouraging research participation, little research has examined different large unconditional incentive amounts. Larger unconditional incentives may result in increased participation of men in psychological research. This randomised study within a case–control trial of men diagnosed with early-stage prostate cancer aimed to investigate whether (a) response rates to a 30-min questionnaire completed via mail, online, or phone would vary with different unconditional incentive amounts, and (b) demographics would vary in those who responded within the different incentive groups.

**Methods:**

We conducted this randomised study within a case–control cross-sectional study aiming to identify the social-ecological factors influencing treatment discontinuation in prostate cancer patients. A total of 238 participants from the cross-sectional study were randomised to receive one of two unconditional incentives (*n* = 121 received AUD$10, *n* = 117 received AUD$20) with the study materials (consent form and survey).

**Results:**

Overall, 113 (47%) responded; *n* = 61/121 (50.4%) in the AUD$10 group, and *n* = 52/117 (44.4%) in the AUD$20 group. No evidence of a difference was found in response rates by incentive group (odds ratio 1.27, 95% CI = 0.76–2.12, *p* = 0.36). Additionally, there were no evident differences in the demographics of the responders vs. non-responders within each incentive group (all *p* > 0.05).

**Conclusions:**

Unlike previous research, we were unable to show that higher monetary incentives were more effective for increasing response rates. An AUD$20 unconditional incentive may be no more effective than a lesser amount for encouraging prostate cancer survivors to participate in research involving long questionnaires. Future research should consider the cost-benefits of providing large unconditional incentives, as non-responses will result in lost resources perhaps better utilised in other engagement strategies.

## Background

Prostate cancer is responsible for a large burden of disease worldwide [1]. It is highly prevalent and associated with significant and long-term morbidity [2]. To ensure high-quality care for patients, and thus reduce disease burden, a greater understanding of patient experiences and unmet needs is essential, especially from the patient perspective. In recent years, psychological studies focusing on patient reported outcomes, such as anxiety and quality of life, have been encouraged in order to inform disease management [3]. However, men are often viewed as a difficult group to engage in psychological research [4], particularly in psycho-oncology [5–8]. While emerging evidence suggests that men with prostate cancer frequently experience unmet physical, social, and informational supportive care needs [9, 10], much of the research to date is qualitative or has relatively small or unrepresentative samples (e.g., recruited participants from only one clinic/hospital). Men have varying health-related needs and preferences [11] and high response rates and representative samples are essential to reflect this variability.

Research into understanding cancer patients’ supportive care needs and experiences has traditionally relied on participant completion of self-reported validated questionnaires [5, 10]. A number of strategies that have been shown to generally improve response rates in paper-based survey research may also be beneficial in recruiting men to these types of studies. Incentives are a potentially important area to investigate in regard to improving recruitment rates in men’s supportive care survey studies. In a large systematic review by Edwards et al. [12] (*N* = 481 trials) that evaluated the effects of 110 different strategies on response rates to postal surveys, odds of response were significantly higher when monetary incentives were utilised, compared to offering no incentive (odds ratio (OR) 1.87; 95% CI 1.73–2.04). Of the 481 randomised controlled trials included, 94 (involving 160,004 participants) evaluated the effect of a monetary incentive. Monetary incentives can either be conditional on response (e.g., mailed out to the participant after they submit a completed survey), or unconditional (e.g., mailed out to the participant with the study materials). Unconditional (versus conditional or non-monetary) monetary incentives have been shown to be the most effective for increasing response rates across a range of populations [12, 13]. Edwards et al. found the odds of postal response increased when unconditional monetary incentives are provided (OR 1.61, 95% CI 1.36–1.89), compared to using conditional monetary incentives [12]. However, there is currently limited guidance for researchers on what amount constitutes an effective monetary incentive, and whether this varies by factors such as participation burden and participant characteristics. Social exchange theory posits the level of monetary incentive needs to be weighted against the burden of the task [14]. If a research incentive is perceived as too high, the participant may be more likely to view it as an economic exchange (rather than a social exchange), resulting in a reduced likelihood of response [14]. Though Edwards et al. [12] did find that responses to postal surveys are slightly higher when a larger incentive is used (odds ratio 1.26, 95% CI 1.14–1.39), much of the research to date has compared conditional and unconditional incentive amounts of around AUD$10 or less, or outcomes using different incentive types (such as monetary amounts versus lottery-style prize draws).

Additionally, emerging research suggests gender differences for monetary incentives in response rates may exist. In a Canadian study by Boulianne [15], men were more responsive to a web-based survey on community attachment and engagement when provided a higher unconditional incentive (CAD$10, equivalent to AUD $10 at the time of the Boulianne study), and women were more responsive with a lower unconditional incentive (CAD$5). However, participants in this study were first-year university students, and these incentive amounts may not be sufficient for paper-based questionnaires of significantly longer length containing personal, health-related questions. Little research has compared larger unconditional monetary incentives (e.g., AUD$10 and over) [16–18], especially in predominantly male cancer populations [15, 19].

We aimed to evaluate the effect of offering different unconditional incentive amounts on response rates in a case control study of men diagnosed with prostate cancer. In particular, we aimed to determine whether (a) response rate would vary by different relatively large unconditional incentive amounts, and (b) patient characteristics (e.g., age, marital status, employment status, education level) would vary in those who responded within the different incentive groups.

## Methods

### Study setting & procedure

We conducted a randomised study within a case–control cross-sectional study aiming to explore the social-ecological reasons why prostate cancer patients discontinued active surveillance without evidence of disease progression [20]. Conducting trials within other research studies is a recognised method for increasing evidence-based knowledge and evaluating or exploring the effectiveness of various approaches to conducting research in a resource efficient way [21]. This sub-study is linked to recruitment for the case–control study. Recruitment was intended to occur through two state-based prostate cancer registries in South Australia and Victoria. Our target sample size for the case–control trial was 450 participants (i.e., 90 case–control groups). Using registry data, men were pre-identified as ‘cases’ (those who had received curative treatment without evidence of disease progression according to predefined criteria) or ‘controls’ (those still on active surveillance or those who had received treatment with signs of disease progression, as clinically recommended) and matched on a 1:4 ratio. The matching ratio was based on the assumption that the response rate among controls would be lower (estimated as 50%) than cases (estimated as 75%), and that a ratio of 1:4 for our sample size would give a probability of 0.94 of having at least 1 of 4 controls for each case.

Unfortunately, due to COVID-19, we were unable to conduct the Victorian arm of the study. As such, all potential participants were contacted through the South Australian Prostate Cancer Clinical Outcomes Collaborative (SA-PCCOC), which captures approximately 90% of newly diagnosed prostate cancer patients in South Australia every year [22]. Recruitment involved SA-PCCOC mailing study materials on our behalf. The study materials included an information statement, consent form, a hard-copy of the survey and a return envelope. An eligibility form was also included. Based on the social-ecological model [23], the survey consisted of 18-pages incorporating validated and researcher-devised measures. Participants could complete and return a hard copy of the survey, access an online version by typing in a link noted on the study materials or call the research team to complete it over the phone. This was to accommodate participant preferences and access needs. A pilot test (*N* = 32 controls) was conducted to assess the probable response rate to the research participation request. Six of the 32 responded (19% response rate). Therefore, alterations to the materials and protocol were made in an attempt to boost the response rate. As recommended by Edwards et al. [12], we reduced the survey length (by two pages), sent all participants a priming letter two weeks prior to study materials, and provided unconditional incentives in the form of a gift card redeemable at thousands of Australian stores (either AUD$10 or AUD$20). Giftcard allocation was randomised. The survey took approximately 30 min to complete. Participants who had not responded after two weeks were mailed a reminder letter. The main study was registered on ANZCTR in February 2020 (trial #12,620,000,170,921), and this sub-study was registered retrospectively in March 2022 (trial 12,622,000,556,741).

### Sample size

Based on the target sample for the main study and the expected effective sizes of unconditional incentives [12] we anticipated we would have reasonable power (> 80%) to detect expected differences in response rates (OR 1.9) between the two groups. However, we were unable to recruit sufficient numbers to the main trial. With Victoria having approximately four times the population of South Australia we anticipated recruiting approximately 75% of our sample from Victoria. With the South Australian registry only, we were only able to invite 270 potential participates to complete the study (consisting of 54 case–control groups).

### Participants

Participants were 18 + years old, had been diagnosed with prostate cancer between January 2014 and October 2019, were able to communicate in English, and had been on active surveillance for at least six months immediately following their prostate cancer diagnosis.

### Randomisation

The allocation sequence was generated by the study statistician (AV) who was blinded to the study participants. The randomization was clustered by the main study case–control group (excluding 32 controls who participated in the pilot), with clusters being block randomized using random block length 2 or 4. Of the 238 participants invited to participate, 121 were allocated the AUD$10 incentive and 117 were allocated the AUD$20 incentive.

### Outcome measures & data collection

The primary outcome was the proportion of responders. Responders were defined as those who either (a) completed and returned a survey (i.e., participants) or (b) did not complete and return the survey but did complete and return a form that had been included with the survey on which individuals could indicate their ineligibility for the case–control study due to having never been on active surveillance (“Never on Active Surveillance” form). Packages returned to sender and returned blank questionnaires were not counted as responses. The secondary outcome was differences in demographic variables in responders within each incentive group. This self-reported information was sourced from the completed surveys (marital status, employment status, and education level) and the SA-PCCOC registry (postcode, diagnosis information, and age). Survey data were collected via mail, online or phone in February – March 2020, and is available on Figshare [24]. Data collected by mail and phone was entered into RedCap, a secure, web-based software platform [25] that also hosted the online version of the survey. Information recorded regarding surveys sent, received, reminders sent, and responses were tracked in Excel and RedCap on a secure University of Adelaide network.

### Blinding

Participants were not advised of the differing incentive amounts included in the survey packages. Author MM was not blind to conditions after group allocation, as she was responsible for facilitated recruitment, material disseminatation, and analysis for both the survey and interviews.

### Statistical methods

Descriptive statistics to illustrate participant demographics were performed. Mixed-effects logistic regression with matched groups as the random effect was used to compare differences in response rates between the two incentive groups. To compare differences in demographics by incentive group responders, Pearson’s chi-square analyses (for categorical variables) and Welch’s two-sample T tests (for continuous variables) were used. The significance level was set at 0.05 (two-sided). All analyses were completed in R [26].

## Results

### Response rate

**Fig. 1:**
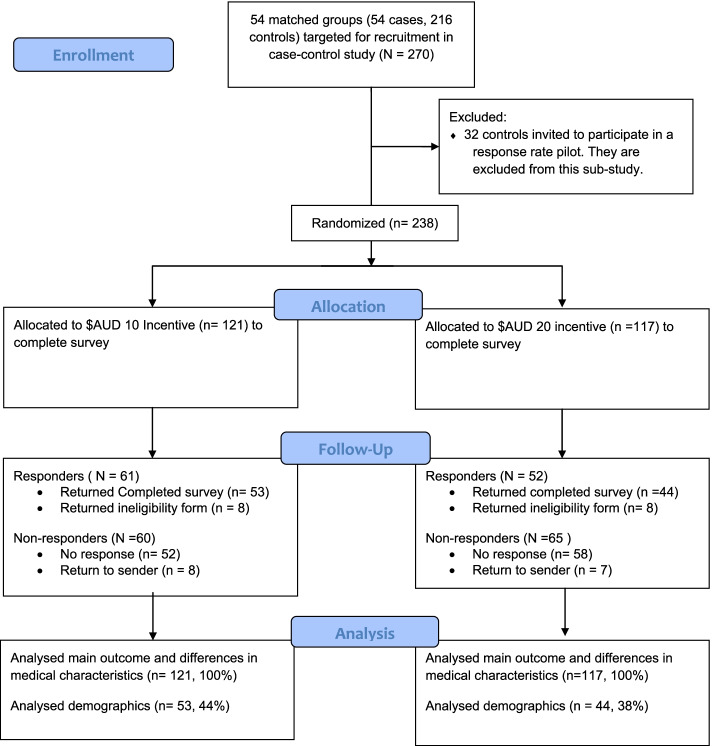
CONSORT diagram of recruitment

A CONSORT diagram of the recruitment process is shown in Fig. 1. In brief, 238 participants from the SA-PCCOC registry were invited to participate in the current study. A total of 113 (47%) responded, with 97 completing and returning a valid survey and a further 16 responding to report that they were ineligible for the study as they had never been on active surveillance.

### Responders

Demographic information on the responders, by incentive group, is presented in Table 1. This information on non-responders was not available, as it was collected within the survey. Clinical characteristics of all randomised participants (*N* = 238), sourced from the SA-PCCOC registry, are shown in Table 2. The average eligible responder was 64 years old, married/partnered (84%), not currently working (71%), had completed post-high school education (69%), and lived in a major South Australian city (71%).

**Table 1:** Demographics of eligible^a^ responders

Demographic Variable	All eligible responders (*n* = 97)	$10 eligible responders (*n* = 53)	$20 eligible responders (*n* = 44)	*p*-value
Age: M (Sd)				
*Mean age in years*	64.4 (6.7)	65.7 (6)	62.8 (7.3)	0.55
Current Treatment Status: N (%)				0.64
*Underwent curative treatment*	51 (52.6)	29 (54.7)	22 (50)	
*On active surveillance or ceased all treatment*	46 (47.4)	24 (45.3)	22 (50)	
Relationship status: N (%)				0.13
*Partnered/married*	81 (83.5)	47 (88.7)	34 (77.3)	
*Single/divorced/widowed*	16 (16.5)	6 (11.3)	10 (22.7)	
Employment: N (%)				0.38
*Currently working (full- or part-time or self-employed)*	28 (28.9)	16 (30.2)	17 (38.6)	
*Not in paid work (e.g. retired, unemployed)*	69 (71.1)	37 (69.8)	27 (61.4)	
Highest Education: N (%)				0.34
*Primary or high school*	29 (29.9)	18 (34.0)	11 (25.0)	
*Post-high school*	67 (69.1)	35 (66.0)	33 (75.0)	
Location^b^: N (%)				0.75
*Major city*	69 (71.1)	37 (69.8)	32 (72.7)	
*Regional or remote area*	28 (28.9)	16 (30.2)	12 (27.3)	
Time Since Diagnosis: M (Sd)				
*Mean years since diagnosis*	2.9 (1.2)	2.8 (1.1)	3.1 (1.3)	0.15

^a^This table only includes eligible responders, as ineligible responders (i.e., the *N* = 16 who completed the “Never on Active Surveillance” form) were not asked to provide demographic information

^b^Location is determined by residential postcode and classified using the Australian Statistical Geographical Classification – Remoteness Area framework [27]

**Table 2:** SA-PCCOC Patient Information for all randomised participants

	**All Participants (*N* = 238)**	**All Responders** **(*N* = 113)**	**All Non-responders (*N* = 125)**
Age at diagnosis: M (Sd)			
*Mean age (years)*	64 (7.3)	64.8 (6.6)	63.4 (7.8)
Current Treatment Status: N (%)			
*Underwent curative treatment*	98 (41.2)	46 (40.7)	52 (41.6)
*On active surveillance or ceased all treatment*	140 (58.8)	67 (59.3)	73 (58.4)
Time Since Diagnosis: M (Sd)			
*Mean years since diagnosis*	3.1 (1.3)	2.9 (1.2)	3.2 (1.4)
Time on active surveillance: M (Sd)			
*Mean months on active surveillance*	22.7 (13)	22.8 (13.5)	22.7 (12.6)

### Difference in responses between AUD$10 and $20 incentives

In the AUD$10 group, *n* = 61/121 (50.4% response rate) responded, and *n* = 52/117 (44.4%) responded in the AUD$20 group. There was no significant difference in response rates to the different incentives (OR = 1.27, 95% CI = 0.76 – 2.12, *p* = 0.37).

### Demographic differences between incentive groups

Respondents allocated to the AUD $20 incentive reported higher rates of being single or divorced and higher rates of post-high-school education compared to respondents to the AUD $10 incentive. However, no statistically significant differences in any of the demographic and health variables (age at diagnosis, marital status, employment status, education level, region/location, and days since diagnosis) were observed between responders to the two different incentives (all *p* > 0.05).

## Discussion

In order to produce generalisable research that is demographically and clinically representative of the target population, researchers must use effective recruitment strategies to ensure a high response rate [12]. Offering unconditional monetary incentives can significantly increase response rates across a range of populations [12]. This trial attempted to incentivise survey participation by men diagnosed with prostate cancer, as they are generally an under-represented cohort in mixed-gender psycho-oncology research due to low response rates [4–8]. This study evaluated the impact of two different unconditional incentive amounts (AUD$10 versus AUD$20) on response rates to a lengthy, personal, health-related questionnaire (when used in conjunction with pre-notification and follow-up). The response rate was approximately 6% higher in the $AUD 10 unconditional incentive group than in the $AUD 20 unconditional incentive group. In line with Social Exchange Theory, this may suggest that the $AUD 20 unconditional incentive was perceived as too high. However, the difference was not statistically significant. Unfortunately, the study likely wasn’t powered to detect differences of this magnitude, which makes the null findings difficult to interpret. These findings are in contrast to previous research suggesting that higher monetary incentive amounts result in higher response rates, though that research was primarily evaluated lower incentive amounts (i.e., under $10AUD), and was not specific to male cancer survivors [12]. It is also noteworthy that our overall response rate of 47% was lower than previous studies that have recruited prostate cancer patients from SA-PCCOC [28], and other research investigating conditional versus unconditional response rates in prostate cancer patients [19]. This may be due to the fact that participation in this study may have involved greater burden (16 written pages total survey, approximately 30 min to complete, including personal questions on mental and physical health) than in many other studies. Data collection also overlapped with the beginning of the COVID-19 pandemic in Australia, which also may have impacted response rates.

Overall, this study found an AUD$20 unconditional incentive was not superior to a AUD$10 unconditional incentive for increasing response rates to a relatively long questionnaire on cancer experiences and unmet needs in prostate cancer survivors. Observational research intending to offer incentives to boost participation rates must also consider the cost benefit of the strategy. If incentives are unconditional, as in the present study, non-responses will result in lost funds perhaps better spent on other effective engagement strategies. Edwards et al. (2009) found that odds of response were significantly higher when strategies such as pre-notification of the study (OR 1.45; 95% CI 1.29–1.63) and follow-up contact (1.35; 95% CI 1.18–1.55) were used. Whilst these strategies were utilised in the present study, we are unable to determine their effect on response rates as these were used with all participants. Future research may consider exploring this and other strategies previously found to be effective, such as providing another copy of the questionnaire when attempting to follow up non-responders [12]. Where sample sizes allow, studies may also consider utilising a factorial design, which would enable analysis of the individual and interactive effects of different strategies [29].

## Conclusions

Conducting trials within studies is a recognised method for identifying effective procedures in the conduct of research (such as the effectiveness of engagement strategies) [21]. In line with social exchange theory, future research should consider whether engagement strategies are balanced to the required tasks in order to be effective. Despite our relatively small sample of prostate cancer survivors, the results suggest that larger monetary unconditional incentives (i.e., over $10) may not be superior to lower incentive amounts (i.e., $10 or less) in this population. Monetary savings by using equally effective smaller incentives would allow valuable resources to be utilised on other strategies to increase engagement and responses in psycho-oncology research. Further research may be needed to generalise these findings to populations not represented in our sample (e.g., prostate cancer patients with metastatic disease).

## Abbreviations

OR: Odds ratio
SWAT: Study within a trial
SA-PCCOC: South Australian Prostate Cancer Clinical Outcomes Collaborative
